# Rational guidewire use in the coronary chronic total occlusion interventions

**DOI:** 10.1186/s43044-020-00115-8

**Published:** 2020-11-07

**Authors:** Ahmet Karabulut, Sevket Gorgulu, Tanıl Kocagoz

**Affiliations:** 1 Department of Cardiology, Acibadem Mehmet Ali Aydınlar University School of Medicine, Acibadem Atakent Hospital,Turgut Ozal Bulvarı, No: 16, 34303 Istanbul, Turkey; 2 Department of Medical Biotechnology, Acibadem Mehmet Ali Aydınlar University School of Medicine, Istanbul, Turkey; 3 Department of Cardiology, Acibadem Kocaeli Hospital, Kocaeli, Turkey

**Keywords:** Affordability, Coronary chronic total occlusion, Clinical practice, Guidewires, Percutaneous coronary interventions

## Abstract

**Background:**

Procedures for coronary chronic total occlusion (CTO) are still a clinical challenge with relatively lower success rates. Recent advances in the biotechnology and introduction of CTO-dedicated guidewires have increased the procedural success rate of CTO interventions. Herein, we aimed to reveal the clinical and angiographic predictors of the crossability of the initial guidewire choice and rational guidewire usage in CTO interventions. A total of 177 patients with an indication for a coronary CTO procedure were included in this study. The use of 1–3 guidewires and crossing of the CTO lesion with the initial guidewire choice was defined as rational guidewire usage. The CTO lesions were classified according to the Japanese chronic total occlusion registry (J-CTO) and EuroCTO scores for evaluating the difficulty of the procedures. Then, a statistical analysis was performed to assess the initial guidewire choice, crossability, and contributors to rational guidewire usage.

**Results:**

The mean J-CTO score was 1.42 ± 1.16, and the mean EuroCTO score was 1.44 ± 1.18. The success rate of the procedures was 90.4%. The initial guidewire choice crossed the lesion in 44.1% of the cases, in which 1–3 guidewires were used (82.1%). The crossability of the polymeric and moderate stiff tip guidewires was higher (82.1% and 64.1%, respectively), and the Pilot series was the most successful brand (36.2%). Logistic regression analysis confirmed that J-CTO score, procedural technique, guidewire type, and stiffness of the tip were the major predictors of rational guidewire usage.

**Conclusion:**

Our analysis showed that the use of polymeric and moderate stiff tip guidewires, particularly the Pilot brand, were associated with rational guidewire usage in easy and intermediate difficulty CTO cases.

## Background

Chronic total occlusions (CTO) are one of the most challenging targets of percutaneous coronary interventions (PCI). Compared to non-CTO PCI, the procedural success rate is lower, and it has greater complication rates, more radiation exposure, and longer procedural times due to its complexity [1, 2].

The presence of CTO was the strongest independent predictor of incomplete PCI in patients with multivessel diseases, which is associated with a worse prognosis [2]. With the conventional PCI strategy, the success rate of CTO interventions is relatively lower compared to standard coronary lesions [3]. The success rate can be as low as 50%, especially in older lesions with previous intervention. The cornerstone manoeuver in CTO interventions is the penetration of the CTO segment with a 0.014 inch guidewire [4–6]. This step is crucial for successful CTO interventions, and this step mainly depends on the choice of guidewire. There have been recent brilliant innovations in the dedicated guidewires for CTO lesions [7–10]. However, the release of new technologic guidewires does not mean the optimal use of new technology in clinical practice under real-world circumstances. Although newly introduced guidewires are smarter and facilitate guidewire penetration, their clinical usage usually varies according to the operator and clinical center. Availability, higher cost, and routine practice of the operator are the major determinants of guidewire preference. The guidelines usually do not define optimal guidewire usage, and the opinions of experts are usually the main factor in the preference of coronary guidewires.

Herein, we aimed to investigate the crossability and rational use of coronary guidewires in CTO interventions. We also aimed to assess the availability and preference of newly released technologic guidewires. We also discussed the clinical and angiographic determinants of the crossability of the initial guidewire choice and its clinical implications.

## Methods

### Patient selection

The investigation is a multicenter, cross-sectional, retrospective-prospective, and observational analysis of subsequent coronary CTO procedures. Between January 1, 2019, and April 30, 2020, a total of 177 patients with an indication for a coronary CTO procedure were included in this study. All the coronary CTO procedures included in this study were performed by the same CTO team. Since our primary endpoint was the evaluation of the biomaterials used in the procedure, a retrospective analysis of the cases with incomplete recordings and lack of clinical and laboratory data were excluded from this study.

### Protocol and procedure

Upon admission, patients were evaluated with anamnesis and physical examination. Then, blood samples were taken for laboratory analysis. Echocardiography was performed before the procedure, and hypokinesia in the territory of the CTO lesion was assessed in detail. Images of each CTO procedure were evaluated, and the Japanese chronic total occlusion registry (J-CTO) score and EuroCTO score of each lesion were calculated according to previously defined criteria [11]. All the biomaterials, including catheters, guidewires, balloons, and stent materials, were analyzed extensively. The preferred techniques in the procedure were also recorded. The number of guidewires, initial choice of guidewire, and final guidewire that crossed the lesion were noted. The amount of contrast, duration of the procedure, and duration of fluoroscopy were also recorded for each procedure. Determinants of the crossability of the initial guidewire choice were evaluated with further statistical analysis. The selection of guidewires was made according to lesion characteristics, availability of the guidewire brands, and preference of the operator.

### Definition

CTO was defined as total occlusion of one major epicardial coronary vessel of at least 3 months duration or undetermined time. The complexity of the CTO lesion and difficulty of the procedure were analyzed according to the J-CTO and EuroCTO scores. A score of 0 was accepted as an easy case, a score of 1 was defined as an intermediate case, a score of 2 was defined as a difficult case, and a score ≥ 3 was accepted as a very difficult case.

Guidewires were classified according to their coatings and tip stiffness. A tip weight load < 1 g was accepted as a soft guidewire, while a tip weight load ≥ 9 g was defined as a stiff guidewire. With regard to tip coatings, the guidewires were classified as polymeric or non-polymeric. The number of 0.014 inch guidewires used was classified as optimal [1–3], acceptable [4–6], overuse [7–9], and overmuch (≥ 10).

Simple crossing of the CTO segment was not defined as a successful procedure. Stent implantation with a distal thrombolysis in myocardial infarction (TIMI) flow of 2–3 was defined as a successful CTO procedure.

### Statistical analysis

Statistical analyses were performed using SPSS 21.0 software (SPSS Inc., Armonk, NY, USA). Mean ± standard deviation, median, and maximum-minimum were used for continuous variables, while percentages were used for categorical variables. Student’s *t* test and one-way ANOVA test were used for evaluating continuous variables between groups. Categorical variables were compared using the chi-square test and Fisher’s exact test. Preference for the guidewires was analyzed according to the procedural technique and the J-CTO and EuroCTO scores. Pearson and Spearman correlation analyses were performed to define the determinants of crossability of the initial guidewire choice. Finally, univariate and multivariate regression analyses were performed to analyze the independent predictors of crossability of the initial guidewire choice. The baseline variables with evident significance (*p* < 0.10) found by univariate analysis were included in the multivariate logistic regression analysis. The results of the model were reported as 95% confidence intervals (CIs) and *p* values. All *p* values were two-sided in the tests, and *p* values < 0.05 were considered to be statistically significant.

### Ethical concern

This study was approved by the Ethics Committee of the Acibadem Mehmet Ali Aydinlar University (date: December 29, 2019; decision number: 2019-20/22).

## Results

The baseline demographic and clinical characteristics of the patients are summarized in Table 1. The procedural success rate was 91.4%. The lesion could not be crossed in 17 cases, which were recorded as failed procedures. The mean age of patients was 62.4 ± 10.5 years, and 141 patients (79.7%) were male. The prevalence of HT and DM were 54.2% and 37.3%, respectively. Nearly 30% of the cases had an anamnesis significant for previous MI. A previous history of CABG was reported in 18.1% of the cases. Approximately three-fourths of the patients presented with stable angina pectoris, and half of the patients showed segmental left ventricular wall hypokinesia.

**Table 1:** Baseline demographic characteristics, clinical features, and laboratory results of the patients. Angiographic, technical, and procedural features of chronic total occlusion lesions were also summarized in the lower part of the table

Variables	% (***n***: 177)	Variables	% (***n***: 177)
Age (years)	62.4 ± 10.5	Stable angina pectoris	75.7% (134)
Range	29–86 years	CCS class 1	4.5% (8)
Sex		CCS class 2	31.6% (56)
Male	79.7% (141)	CCS class 3	35.6% (63)
Female	20.3% (36)	CCS class 4	*4% (7)*
Presence of diabetes mellitus	*37.3% (57)*	Unstable angina	4.5% (8)
Presence of hypertension	54.2% (96)	Previous MI	29.3% (52)
Presence of dyslipidemia	43.5% (77)	Previous CABG	18.1% (32)
Smoking		Documentation of ischemia	29.9% (53)
Current smoker	32.2% (57)	LV EF	
Ex smoker	20.3% (36)	> 50%	66.7% (118)
BMI (kg/m^2^)	28.27 ± 4.42	35–50%	28.8% (51)
Initial creatinine (mg/dl)	1.00 ± 0.33	< 35%	4.5% (8)
Post-procedural creatinine (mg/dl)	1.04 ± 0.34	Hypokinesia in cto territory	50.3% (89)
Initial GFR (ml/minutes)	90.3 ± 31.8	Akinesia in cto territory	2.3% (4)
Vessel		J-CTO Score	1.42 ± 1.16
LAD	32.8% (58)	0	26.0% (46)
LCX	19.8% (35)	1	28.2% (50)
RCA	47.5% (84)	2	27.7% (49)
Segment		3	*13.6% (24)*
Ostial	*6.8%* (12)	4	4.0% (7)
Proximal	36.7% (65)	5	0.6% (1)
Mid	46.3% (82)	EuroScore	1.44 ± 1.18
Distal	10.2% (18)	0	23.2% (41)
In-stent occlusion	13% (23)	1	31.1% (55)
Multilevel occlusion	11.9% (21)	2	32.8% (58)
Bifurcation in cto segment	33.9% (60)	3	*6.8%* (12)
Radial access	58.8% (104)	4	4.0% (7)
Contra-lateral injection	53.1% (94)	5	1.7% (3)
Retrograde approach	4% (7)	6	0.6% (1)
Guiding catheter		Procedural technique	
6F	16.9% (30)	Single-wire cross	60.5% (107)
7F	83.1% (147)	Parallel-wire cross	6.2% (11)
Successfull procedure	90.4% (170)	Step up—step down	23.2% (41)
		Dissection and re-entry	8.5% (15)
		Reversed cart	1.7% (3)

*BMI* body mass index, *GFR* glomerular filtration rate, *CCS* Canadian Cardiovascular Society, *MI* myocardial infarction, *CABG* coronary artery bypass grafting, *LV EF* left ventricular ejection fraction, *LAD* left anterior descending artery, *LCX* left circumflex artery, *RCA* right coronary artery, *F* French, *J-CTO* Japan registry of chronic total occlusion

Angiographic and procedural features of the CTO lesions are also summarized in Table 1. The right coronary artery was the most common vessel having a CTO segment (47.5%). The middle and proximal segmental locations were more common compared to the distal and ostial locations. The lesion characteristics including bifurcation, in-stent occlusion, multivessel occlusion that aggravated the difficulty of the procedure was < 50%. The J-CTO and EuroCTO scores revealed that 55% of the lesions were of either easy or intermediate difficulty, and the number of very difficult cases was definitely low (18.2% of the cases). Single-wire crossing came to the forefront as a major procedural technique, followed by the step up-step down strategy (60.5% and 23.2%, respectively).

Guidewire choice and their characteristics are summarized in Table 2. The average number of guidewires used was 4.32 ± 2.39, and in 47.5% of the cases, 1–3 guidewires were used during the procedure, which was described as a rational use of guidewires. The initial guidewire choice was usually the polymeric type, and the Fielder and Pilot brands were the most commonly preferred guidewires (26.6% and 36.2%, respectively). Moderate and soft tip load guidewires were preferred over stiff tip guidewires as a first choice (moderate tip load: 55.4% and stiff tip load: 12.4%). Stiff tip and non-polymeric guidewires were more frequently preferred as a final guidewire compared to the initial choice. The Pilot, Fielder, and Gaia brands were the most successful guidewires in terms of crossing the CTO segment. Although the Gaia brand was not a favorite for the initial guidewire choice, it was the final choice in 13% of the cases.

**Table 2:** Type and structural features of 0.014 inch coronary guidewires used for chronic total occlusion procedure

Variables	% (***n***: 177)	Variables	% (***n***: 177)
Guidewire number	4.32 ± 2.39	Final guidewire	
1–3	47.5% (84)	Fielder brand	22.6% (40)
4–6	36.7% (65)	Miracle brand	5.1% (9)
7–10	12.4% (22)	Pilot brand	26.6% (47)
> 10	3.4% (6)	Gaia brand	*13%* (23)
Initial guide wire		Confianza brand	9% (16)
Fielder brand	26.6% (47)	Ultimate brand	1.7% (3)
Miracle brand	6.2% (11)	Sion brand	2.3% (4)
Pilot brand	36.2% (64)	PT brand	1.7% (3)
Gaia brand	2.3% (4)	Gladius brand	5.6% (10)
Confianza brand	11.3% (20)	Progress brand	2.3% (4)
Ultimate brand	6.8% (12)	Cross-it brand	*0.6%* (1)
Sion brand	5.6% (10)	Failed procedure	*9.6%* (17)
PT brand	2.3% (4)	Final guidewire type	
Gladius brand	2.3% (4)	Polymeric	56.5% (100)
Progress brand	0.5% (1)	Non-poylmeric	*33.9% (60)*
Initial guidewire type	16.9% (30)	Final guidewire stiffness	
Polymeric	67.2% (119)	Soft (< 1 gram)	24.9% (44)
Non-poylmeric	32.8% (58)	Moderate (1–9 g)	47.5% (84)
Initial guidewire stiffness		Stiff (≥ 9 g)	18.1% (32)
Soft (< 1 g)	32.2% (57)	Same initial—final guidewire	44.1% (78)
Moderate (1–9 g)	55.4% (98)		
Stiff (≥ 9 g)	12.4% (22)		

The crossability of the initial guidewires and their clinical correlation are summarized in Tables 3 and 4. Initially, the preferred guidewire crossed the lesion in 78 patients (44.1%). Polymeric and moderate stiff tip guidewires had superior crossability. Pilot 200, Pilot 50, and Fielder XT had higher crossability rates as a first-choice guidewire (30.8%, 20.5%, and 20.5%, respectively). Even within the same brand, there were opposing results. Contrary to the Fielder XT, the crossability of the Fielder FC was not good (1.3% vs 7.1%, respectively). This result might indicate that distal tip composition is crucial to crossability. Within the non-polymeric groups, Confianza 9 had a relatively higher likelihood of lesion penetration as an initial guidewire choice.

**Table 3:** Clinical and angiographic predictors of first-choice guidewire and it’s crossability through the CTO segment

Variables	Same initial-final guidewire (+)	Same initial-final guidewire (−)	***p***
Age (years)	63.41 ± 10.9	61.74 ± 10.2	0.30
Sex			0.07
Male	71.8% (56)	84.8% (84)	
Female	26.9% (21)	15.2% (15)	
BMI (mg/dl)	27.9 ± 4.65	28.5 ± 4.23	0.42
Initial creatinine (mg/dl)	0.96 ± 0.30	1.04 ± 0.36	0.12
GFR (ml/minutes)	64.2 ± 22.6	68.7 ± 21.3	0.76
LV EF			0.07
> 50%	73.1% (57)	61.6% (61)	
35–50%	20.5% (16)	35.4% (35)	
< 35%	6.4% (5)	3.0% (3)	
Hypokinesia in cto territory	41% (32)	57.6% (57)	0.09
Procedural technique			< 0.01
Single-wire cross	88.5% (69)	38.4% (38)	
Parallel wire cross	1.3% (1)	10.1% (10)	
Step up—step down	5.1% (4)	37.4% (37)	
Dissection and re-entry	5.1% (4)	11.1% (11)	
Reversed cart	0% (0)	3% (3)	
Radial access	55.1% (43)	61.6% (61)	0.38
Kontralateral contrast injection	48.7% (38)	56.6% (56)	0.29
J-CTO Score	0.83 ± 0,88	1.89 ± 1.13	< 0.01
0	42.3% (33)	13.1% (13)	
1	37.2% (29)	21.2% (21)	
2	16.7% (13)	36.4% (36)	
3	2.6% (2)	22.2% (22)	
4	1.3% (1)	6.1% (6)	
5	0% (0)	1% (1)	
EuroCTO score	0.93 ± 0.90	1.84 ± 1.23	< 0.01
Target vessel			0.89
LAD	34.6% (27)	31.3% (31)	
LCX	19.2% (15)	20.2% (20)	
RCA	46.2% (36)	48.5% (48)	
CTO segment			0.50
Ostial	5.1% (4)	8.1% (8)	
Proximal	33.3% (26)	39.4% (39)	
Mid	52.6% (41)	41.4% (41)	
Distal	9% (7)	11.1% (11)	
Guidewire number	2.85 ± 1.31	5.48 ± 2.43	< 0.01
Ballon number	2.85 ± 1.44	3.20 ± 1.88	0.18
Stent number	1.58 ± 0.70	1.91 ± 0.91	0.01
Stent length (mm)	50.6 ± 21.8	65.7 ± 33.0	0.001
Fluoroscopy duration	22.2 ± 13.1	45.6 ± 24.4	< 0.01
Contrast amount (ml)	242 ± 114	362 ± 173	< 0.01
Total	44.1% (78)	55.9% (99)	

*BMI* body mass index, *GFR* glomerular filtration rate, *LV EF* left ventricular ejection fraction, *J-CTO* Japan registry of chronic total occlusion, *LAD* left anterior descending artery, *LCX* left circumflex artery, *RCA* right coronary arterY, *CTO* chronic total occlusion

*P < 0.05* is indicated as significant

**Table 4:** Distributions of guidewire type, number, and brands according to crossability of initial choice guidewire

Variables	Same initial-final guidewire (+)	Same initial-final guidewire (−)	***p***
Initial guidewire type			< 0.001
Polymeric	82.1% (64)	55.6% (55)	
Non-polymeric	17.9% (14)	44.4% (44)	
Initial guidewire stiffness			0.07
Soft (< 1 g)	28.2% (22)	35.4% (35)	
Moderate (1–9 g)	64.1% (50)	48.5% (48)	
Stiff (≥ 9 g)	7.7% (6)	16.2% (16)	
Guidewire number			< 0.001
1–3	82.1% (64)	20.2% (20)	
4–6	15.4% (12)	53.5% (53)	
7–10	2.6% (2)	20.2% (20)	
≥ 10	0% (0)	3.4% (6)	
Guidewire brand			0.005
Fielder FC	1.3% (1)	7.1% (7)	
Fielder XT	20.5% (16)	14.1% (14)	
Fielder XT-A	1.3% (1)	3.0% (3)	
Fielder XT-R	5.1% (4)	1.0% (1)	
Miracle 3	1.3% (1)	1.0% (1)	
Miracle 6	3.8% (3)	6.1% (6)	
Miracle 12	0% (0)	0% (0)	
Pilot 50	20.5% (16)	12.1% (12)	
Pilot 150	1.3% (1)	0% (0)	
Pilot 200	30.8% (24)	11.1% (11)	
Gaia 1	0 % (0)	1.0% (1)	
Gaia 2	1.3% (1)	1.0% (1)	
Gaia 3	0% (0)	1.0% (1)	
Confianza 9	7.7% (6)	14.1% (14)	
Sion	1.3% (1)	9.1% (9)	
Sion black	0% (0)	0% (0)	
Ultimate 3	2.6% (2)	10.1% (10)	
PT 2	0% (0)	4.0% (4)	
Gladius	1.3% (1)	3.0% (3)	
Progress 200	0% (0)	1.0% (1)	
Total	44.1% (78)	55.9% (99)	

*P* < 0.05 is indicated as significant

Table 5 reveals the impact of procedural strategy on the guidewire choice. The polymeric Pilot brands were the most common initial guidewire choice in the single-wire cross technique (41.1%). The polymeric Fielder brands were more commonly preferred for the parallel wire technique (45.5%). The non-polymeric Gaia and Confianza brands were preferred in the step up-step down strategy and in the dissection and re-entry technique. Soft guidewires were the dominant choice for the retrograde approach.

**Table 5:** Distribution of first-choice and final crossing guidewires characteristics according to a procedural technique

Variables	Single wire	Parallel wire	Step up Step down	Dissection re-entry	Reversed cart	***p***
Guidewire number	3.63 ± 2.23	4.27 ± 1.19	5.36 ± 2.26	5.80 ± 2.56	7.66 ± 2.08	< 0.001
1–3	65.4% (70)	36.4% (4)	19.5% (8)	13.3% (2)	0% (0)	
4–6	24.3% (26)	63.6% (7)	53.7% (22)	60.0% (9)	33.3% (1)	
7–10	7.5% (8)	0% (0)	24.4% (10)	20.0% (3)	33.3% (1)	
> 10	2.8% (3)	0% (0)	2.4% (1)	6.7% (1)	33.3% (1)	
Initial guide wire						0.01
Fielder brand	25.2% (27)	45.5% (5)	24.4% (10)	26.7% (4)	33.3% (1)	
Miracle brand	5.6% (6)	9.1% (1)	7.3% (3)	6.7% (1)	0% (0)	
Pilot brand	41.1% (44)	27.3% (3)	31.7% (13)	26.7% (4)	0% (0)	
Gaia brand	0.9% (1)	0% (0)	2.4% (1)	13.3.% (2)	0% (0)	
Confianza brand	5.6% (6)	9.1% (1)	22.0% (9)	26.7% (4)	0% (0)	
Ultimate brand	7.5% (8)	9.1% (1)	7.3% (3)	0% (0)	0% (0)	
Sion brand	8.4% (9)	0% (0)	0% (0)	0% (0)	33.3% (1)	
PT brand	2.8% (3)	0% (0)	0% (0)	0% (0)	33.3% (1)	
Gladius brand	1.9% (2)	0% (0)	4.9% (2)	0% (0)	0% (0)	
Progress brand	0.9% (1)	0% (0)	0% (0)	0% (0)	0% (0)	
Initial guidewire type						0.57
Polymeric	71.0% (76)	72.7% (8)	61.0% (25)	53.3% (8)	66.7% (2)	
Non-poylmeric	29.0% (731)	27.3% (3)	39.0% (16)	46.7% (7)	33.3% (1)	
Initial wire stiffness						0.01
Soft (< 1 g)	35.5% (38)	36.4% (4)	19.5% (8)	26.7% (4)	100% (3)	
Moderate (1–9 g)	57.9% (62)	54.5% (6)	58.5% (24)	40.0% (6)	0% (0)	
Stiff (≥ 9 g)	6.5% (7)	9.1% (1)	22.0% (9)	33.3% (5)	0% (0)	
Final guidewire						< 0.001
Fielder brand	29.3% (27)	0% (0)	12.5% (5)	57.1% (8)	0% (0)	
Miracle brand	6.5% (6)	9.1% (1)	5.0% (2)	0% (0)	0% (0)	
Pilot brand	40.2% (37)	9.1% (1)	15.0% (6)	21.4% (3)	0% (0)	
Gaia brand	10.9% (10)	27.3% (3)	22.5% (9)	0% (0)	33.3% (1)	
Confianza brand	7.6% (7)	18.2% (2)	17.5% (7)	0% (0)	0% (0)	
Ultimate brand	2.2% (2)	0% (0)	2.5% (1)	0% (0)	0% (0)	
Sion brand	1.1% (1)	9.1% (1)	5.0% (2)	0% (0)	0% (0)	
PT brand	0% (0)	0% (0)	0% (0)	21.4% (3)	0% (0)	
Gladius brand	1.9% (2)	9.1% (1)	12.2% (5)	0% (0)	66.7% (2)	
Progress brand	0% (0)	18.2% (2)	4.9% (2)	0% (0)	0% (0)	
Cross-it brand	0% (0)	0% (0)	2.4% (1)	0% (0)	0% (0)	
Failed procedure	14.0% (15)	0% (0)	2.4% (0)	6.7% (1)	0% (0)	
Final guidewire type						< 0.001
Polymeric	71.7% (66)	18.2% (2)	40.0% (16)	100% (14)	66.7% (2)	
on-poylmeric	28.3% (2)	81.8% (9)	60.0% (24)	0% (0)	33.3% (1)	
Final wire stiffness						0.001
Soft (< 1 g)	29.3% (27)	9.1% (1)	17.5% (7)	64.3% (9)	0% (0)	
Moderate (1–9 g)	57.6% (53)	36.4% (4)	50.0% (20)	35.7% (5)	66.7% (2)	
Stiff (≥ 9 g)	13.0% (12)	54.5% (6)	32.5% (13)	0% (0)	33.3% (1)	
Same initial-final wire	64.5% (69)	9.1% (1)	9.8% (4)	26.7% (4)	0% (0)	< 0.001
Total	60.5% (107)	6.2% (11)	23.2% (41)	8.5% (15)	1.7% (3)	

*g* gram

*P* < 0.05 is indicated as significant

The correlation between procedural difficulty and guidewire choice is revealed in Tables 6 and 7 in terms of CTO score. Both the J-CTO and EuroCTO scores showed similar correlations with guidewire preference and performance. Polymer jacketed guidewires were most commonly used in lesions with CTO scores of 0–1, whereas non-polymeric guidewires were most commonly used in lesions with CTO scores of 3–4. The polymeric Pilot and Fielder brand guidewires were the most common initial guidewire choices. The Confianza brand was the most commonly used non-polymeric guidewire in lesions with CTO scores of 2–4. The average number of guidewires used was also statistically significant in correlation with both the J-CTO and EuroCTO scores. There was a linear correlation between the J-CTO score, EuroCTO score, and guidewire number. More complex cases were associated with a higher number of guidewires (*p* < 0.001). The final guidewire type and stiffness did not differ statistically. However, the polymeric Pilot brands were most successful in lesions with CTO scores of 0–1. The non-polymeric Gaia brand had superior crossability in lesions with CTO scores of 2–4 (*p* = 0.04). The crossability of the initial guidewire choice had a close relationship with both the J-CTO and EuroCTO scores. The same initial and final guidewire was used in 71.7% of lesions with a J-CTO score of 0, which dropped to 14.7% in lesions with a J-CTO score of 4 (*p* < 0.001). This analysis showed that the crossability of the initial guidewires was higher in lesions with J-CTO scores of 0–1 and gradually declined as the score increased.

**Table 6:** Distribution of first-choice and final crossing guidewires characteristics according to J-CTO score

Variables	J-CTO 0	J-CTO 1	J-CTO 2	J-CTO 3	J-CTO 4	J-CTO 5	***p***
Guidewire number	3.21 ± 1.57	3.60 ± 1.88	4.87 ± 2.50	6.04 ± 2.05	7.28 ± 4.02	3.0 ± 1.0	< 0.001
1–3	73.9% (34)	60.0% (30)	32.7% (16)	8.3% (2)	14.3% (1)	100% (1)	
4–6	21.7% (10)	30.0% (15)	49.0% (24)	54.2% (13)	42.9% (3)	0% (0)	
7–10	4.3% (2)	8.0% (4)	14.3% (7)	33.3% (8)	14.3% (1)	0% (0)	
> 10	0% (0)	2.0% (1)	4.1% (2)	4.2% (1)	28.6% (2)	0% (0)	
Initial guide wire							0.12
Fielder brand	34.8% (16)	24.0% (12)	24.5% (12)	29.2% (7)	0% (0)	0% (0)	
Miracle brand	4.3% (2)	6.0% (3)	8.2% (4)	4.2% (1)	14.3% (1)	0% (0)	
Pilot brand	47.8% (22)	44.0% (22)	30.6% (1)	12.5% (3)	28.6% (2)	0% (0)	
Gaia brand	0% (0)	2.0% (1)	2.0% (1)	8.3.% (2)	0% (0)	0% (0)	
Confianza brand	2.2% (1)	6.0% (3)	12.2% (6)	29.2% (7)	42.9% (3)	0% (0)	
Ultimate brand	4.3% (2)	4.0% (2)	12.2% (6)	8.3% (2)	0% (0)	0% (0)	
Sion brand	2.2% (1)	6.0% (3)	8.2% (4)	4.2% (1)	14.3% (1)	0% (0)	
PT brand	0% (0)	4.0% (2)	0% (0)	4.2% (1)	0% (0)	100% (1)	
Gladius brand	2.2% (1)	4.0% (2)	2.0% (1)	0% (0)	0% (0)	0% (0)	
Progress brand	2.2% (1)	0% (0)	0% (0)	0% (0)	0% (0)	0% (0)	
Initial guidewire type							0.001
Polymeric	84.8% (39)	76.0% (38)	57.1% (28)	45.8% (11)	28.6% (2)	100% (1)	
Non-poylmeric	15.2% (7)	24.0% (12)	42.9% (21)	54.2% (13)	71.4% (5)	0% (0)	
Initial guidewire stiffness							0.02
Soft (< 1 g)	34.8% (16)	32.0% (16)	30.6% (15)	33.3% (8)	14.3% (1)	100% (1)	
Moderate (1–9 g)	60.9% (28)	62.0% (31)	55.1% (27)	37.5% (9)	42.9% (3)	0% (0)	
Stiff (≥ 9 g)	4.3% (2)	6.0% (3)	14.3% (7)	29.2% (7)	42.9% (3)	0% (0)	
Final guidewire							0.04
Fielder brand	32.6% (15)	17% (8)	23.8% (10)	31.6% (6)	16.7% (1)	0%(0)	
Miracle brand	4.3% (2)	8.5% (4)	2.4% (1)	10.5% (2)	0% (0)	0% (0)	
Pilot brand	43.5% (20)	38.3% (18)	14.3% (6)	10.5% (2)	16.7% (1)	0% (0)	
Gaia brand	8.7% (4)	12.8% (6)	21.4% (9)	15.8% (3)	16.7% (1)	0% (0)	
Confianza brand	6.5% (3)	12.8% (6)	11.9% (5)	10.5% (2)	0% (0)	0% (0)	
Ultimate brand	4.3% (2)	0% (0)	2.1% (1)	0% (0)	0% (0)	0% (0)	
Sion brand	0% (0)	2.1% (1)	4.8% (2)	5.3% (1)	0% (0)	0% (0)	
PT brand	0% (0)	0% (0)	4.8% (2)	5.3% (1)	0% (0)	0% (0)	
Gladius brand	0% (0)	6.0% (3)	6.1% (3)	8.3% (2)	28.6% (2)	0% (0)	
Progress brand	0% (0)	2.0% (1)	4.1% (2)	0% (0)	14.3% (1)	0% (0)	
Cross-it brand	0% (0)	0% (0)	2.0% (1)	0% (0)	0% (0)	0% (0)	
Failed procedure	0% (0)	6.0% (3)	14.3% (7)	20.8% (5)	14.3% (1)	100% (1)	
Final guidewire type							0.15
Polymeric	76.1% (35)	61.7% (29)	50.0% (21)	57.9% (11)	66.7% (4)	0% (0)	
Non-poylmeric	23.9% (11)	38.3% (18)	50.0% (21)	42.1% (8)	33.3% (2)	0% (0)	
Final guidewire stiffness							0.23
Soft (< 1 g)	30.4% (14)	21.3% (10)	28.6% (12)	36.8% (7)	16.7% (1)	0% (0)	
Moderate (1–9 g)	63.0% (29)	55.3% (26)	42.9% (18)	42.1% (8)	50.0% (2)	0% (0)	
Stiff (≥ 9 g)	6.5% (3)	23.4% (11)	28.6% (12)	21.1% (4)	33.3% (2)	0% (0)	
Same initial-final wire	71.7% (33)	58% (29)	26.5% (13)	8.3% (2)	14.3% (1)	0% (0)	< 0.001
Total	26.0% (46)	28.2% (50)	27.7% (49)	13.6% (24)	4.0% (7)	0.6% (1)	

*J-CTO* Japan registry of chronic total occlusion, *g* gram

*P < 0.05* is indicated as significant

**Table 7:** Distribution of first-choice and final crossing guidewire characteristics according to EuroCTO score

Variables	E-CTO 0	E-CTO 1	E-CTO 2	E-CTO 3	E-CTO 4	E-CTO 5	***p***
Guidewire number	3.29 ± 1.79	3.61 ± 1.70	5.31 ± 2.57	4.66 ± 3.02	5.42 ± 2.43	7.33 ± 4.16	< 0.001
1–3	70.7% (29)	60.0% (33)	27.6% (16)	41.7% (5)	14.3% (1)	0% (0)	
4–6	22.0% (9)	30.9% (17)	46.6% (27)	41.7% (5)	71.4% (5)	66.7% (2)	
7–10	7.3% (3)	9.1% (5)	20.7% (12)	8.3% (1)	0% (0)	0% (0)	
> 10	0% (0)	0% (0)	5.2% (3)	8.3% (1)	14.3% (1)	33.3% (1)	
Initial guide wire							0.06
Fielder brand	31.7% (13)	21.8% (12)	25.9% (15)	41.7% (5)	28.6% (2)	0% (0)	
Miracle brand	4.9% (2)	5.5% (3)	6.9% (4)	8.3% (1)	0% (0)	33.3% (1)	
Pilot brand	48.8% (20)	50.9% (28)	22.4% (13)	8.3% (1)	28.6% (2)	0% (0)	
Gaia brand	2.4% (1)	1.8% (1)	3.4% (2)	0% (0)	0% (0)	0% (0)	
Confianza brand	2.4% (1)	1.8% (1)	22.4% (13)	16.7% (2)	14.3% (1)	33.3% (1)	
Ultimate brand	2.4% (1)	5.5% (3)	10.3% (6)	8.3% (1)	14.3% (1)	0% (0)	
Sion brand	4.9% (2)	5.5% (3)	6.9% (4)	0% (0)	0% (0)	33.3% (1)	
PT brand	0% (0)	1.8% (1)	1.7% (1)	8.3% (1)	14.3% (1)	0% (0)	
Gladius brand	0% (0)	5.5% (3)	0% (0)	8.3% (1)	0% (0)	0% (0)	
Progress brand	2.4% (1)	0% (0)	0% (0)	0% (0)	0% (0)	0% (0)	
Initial wire type							0.001
Polymeric	80.5% (33)	80.0% (44)	50.0% (29)	66.7% (8)	71.4% (5)	0% (0)	
Non-poylmeric	19.5% (8)	20.0% (11)	50.0% (29)	33.3% (4)	28.6% (2)	100% (3)	
Initial wire stiffness							0.01
Soft (< 1 g)	34.1% (14)	25.5% (14)	32.8% (19)	50.0% (6)	42.9% (3)	33.3% (1)	
Moderate1-9 g)	61.0% (25)	70.9% (39)	44.8% (26)	33.3% (4)	42.9% (3)	33.3% (1)	
Stiff (≥ 9 g)	4.9% (2)	3.6% (2)	22.4% (13)	16.7% (2)	14.3% (1)	33.3% (1)	
Final guidewire							0.39
Fielder brand	26.8% (11)	19.6% (10)	26.9% (14)	37.5% (3)	20.0% (1)	0% (0)	
Miracle brand	7.3% (3)	3.9% (2)	5.8% (3)	0% (0)	20% (1)	0% (0)	
Pilot brand	43.9% (18)	39.2% (20)	15.4% (8)	12.5% (1)	0% (0)	0% (0)	
Gaia brand	12.2% (5)	9.8% (5)	21.2% (11)	12.5% (1)	0% (0)	0% (0)	
Confianza brand	7.3% (3)	13.7% (7)	7.7% (4)	12.5% (1)	20.0% (1)	0% (0)	
Ultimate brand	2.4% (1)	3.9% (2)	0% (0)	0% (0)	0% (0)	0% (0)	
Sion brand	0% (0)	2.0% (1)	5.8% (3)	0% (0)	0% (0)	0% (0)	
PT brand	0% (0)	2.0% (1)	5.8% (3)	0% (0)	0% (0)	0% (0)	
Gladius brand	0% (0)	3.6% (2)	6.9% (4)	16.7% (2)	14.3% (1)	33.3% (1)	
Progress brand	0% (0)	1.8% (1)	3.4% (2)	0% (0)	14.3% (1)	0% (0)	
Cross-it brand	0% (0)	0% (0)	1.7% (1)	0% (0)	14.3% (1)	0% (0)	
Failed procedure	0% (0)	7.3% (4)	10.3% (6)	33.3% (4)	28.6% (2)	33.3% (1)	
Final guidewire type							0.50
Polymeric	70.7% (29)	64.7% (33)	53.8% (28)	75.0% (6)	40.0% (2)	50.0% (1)	
Non-poylmeric	29.3% (12)	35.3% (18)	46.2% (24)	25.0% (2)	60.0% (3)	50.0% (1)	
Final wire stiffness							0.20
Soft (< 1 g)	24.4% (10)	19.6% (10)	36.5% (19)	37.5% (3)	20.0% (1)	0% (0)	
Moderate (1–9 g)	65.9% (27)	60.8% (31)	38.5% (20)	37.5% (3)	40.0% (2)	50.0% (1)	
Stiff (≥ 9 g)	9.8% (4)	19.6% (10)	25.0% (13)	25.0% (2)	40.0% (2)	50.0% (1)	
Same initial-final wire	68.3% (28)	58.2% (32)	24.1% (14)	25.0% (3)	14.3% (1)	0% (0)	< 0.001
Total	23.2% (41)	31.1% (55)	32.8% (58)	6.8% (12)	4.0% (7)	1.7% (3)	

*EuroCTO 6 group:* just 1 patient, guidewire number 8, initial wire is non-polymeric, stiff Confianza 9 brand, final wire is polymeric, *soft* Fielder FC brand, *E-CTO* Euro chronic total occlusion

*P* < 0.05 is indicated as significant

### Multivariate analysis

Univariate and multivariate logistic regression analyses were performed to obtain determinants of the crossability of the initial guidewire. The procedural technique and J-CTO score were independent predictors of the crossability of the initial guidewire (OR 0.326, CI 0.199; 0.535, *p* < 0.001 and OR 0.363, CI 0.240; 0.550, *p* < 0.001) (Table 8). A lower J-CTO score was a predictor of the use of fewer guidewires. The initial guidewire type and stiffness were also independent predictors of the success of the initial guidewire (OR 5.763, CI 1.980; 16.775, *p* = 0.001 and OR 0.324, CI 0.149; 0.705, *p* = 0.004). The initial guidewire brand did not have a significant impact on the crossability of the initial guidewire choice (*p* = 0.26).

**Table 8:** Multivariate logistic regression analysis to reveal the determinants of the crossability of initial choice guidewire

Dependant variable: Same initial-final guidewire		Multivariate analysis	
	Odds Ratio	95% CI	*p*
Sex (male/female)	0.312	0.112; 0.869	0.02
LV EF	0.717	0.314; 1.640	0.43
Hypokinesia on cto territory	*0.733*	*0.342; 1.570*	0.42
J-CTO Score	0.363	0.240; 0.550	< 0.001
Procedural technique	0.326	0.199; 0.535	< 0.001
Initial guidewire	0.949	0.866; 1.040	0.26
Initial guidewire type	5.763	1.980; 16.775	0.001
Initial guidewire stiffness	0.324	0.149; 0.705	0.004

LV EF left ventricular ejection fraction, J-CTO Japan registry of chronic total occlusion

*P < 0.05 is indicated as significant*

## Discussion

This study was the first report from Turkey that analyzed rational guidewire use and procedural success in CTO procedures. We found that rational guidewire usage (1–3 guidewires) was possible, especially in easy and moderately difficult CTO procedures. The crossing of the CTO lesion with the initial guidewire choice decreased both biomaterial use and the cost of the procedure. Classification of the CTO lesion by the J-CTO or EuroCTO score led to a more precise strategy. Polymeric jacketed moderate stiff tip guidewires, particularly the Pilot brand, had superior crossability in the easy and moderately difficult CTO procedures.

Cardiac CTO procedures necessitate special instruments to increase the success rate. Recent advances in medical biotechnology have led to the manufacture of smarter coronary guidewires, which directly affect the procedural success of CTO interventions [12–15]. The availability of new generation CTO-specific biomaterials is still a concern in most cardiac centers. Most centers use a few brands of CTO-specific materials, including guidewires, microcatheters, and balloon catheters. Moreover, patients have to pay for a certain percentage of the CTO procedure by themselves because of the limited coverage of insurance companies. So, the rational use of such biomaterials is important for sustainable CTO interventions.

Each CTO-dedicated guidewire has a different tip structure, polymer jacket, and tip stiffness [14]. Variations in guidewire structure affect the steerability, crossability, and tactile feedback of the guidewire. Operators should be familiar with the structure of the guidewires, then they should form a strategy for the technique and guidewire choice [13]. The polymer-jacketed guidewire has superior steerability; however, a subintimal course is common, and due to limited tactile feedback, operators should use them very carefully [7, 16]. Non-polymeric guidewires have a better tactile feedback and intraluminal course. However, their steerability is inferior to polymeric guidewires. A stiff tip is important for lesion penetration and wire escalation. A tough stump necessitates more stiff guidewires. Our experience shows that non-polymeric and stiff guidewires are more commonly preferred in complex lesions. Polymeric guidewires are more useful in easy and intermediate difficulty lesions. The presence of microchannels is the optimal indication for soft and moderate stiff tip polymeric guidewires [17–19].

Lesion characteristics including length, calcification, tortuosity, stump, distal area, and occlusion duration are all important for defining an optimal strategy [18, 19]. Several CTO scores were proposed to determine the success of a CTO procedure [11]. Instead of evaluating the lesion characteristics one by one, simply totalling the CTO scores may predict the success rate of the procedure. The J-CTO score was developed with the help of the Japan Multicentre CTO registry in 2006. It was approved by investigations that predicted the success rate of antegrade CTO interventions [20]. A lower J-CTO score is usually accepted as an easy or intermediate difficulty case. Higher scores indicate very difficult cases. Our procedures were performed mostly by the antegrade approach. Thus, the J-CTO score is a suitable method to assess our cases. Approximately 80% of the patients had lesions with J-CTO scores of 0–2. So, we can conclude that our results are appropriate to analyze easy, intermediate, and difficult cases. Very difficult cases constituted just 17% of the total cases. There was an inverse correlation between the J-CTO score and success rate, which was in accord with the medical literature [20, 21]. All lesions with a J-CTO of 0 had successful interventions. There was also an inverse relationship between the crossability of the initial guidewire choice and J-CTO score. More than 50% of the lesions with J-CTO scores of 0–1 were crossed with the initial guidewire. This statistic reveals the importance of the initial guidewire choice. Reasonable initial guidewire choice and the single-wire cross strategy would lead to shorter procedural and fluoroscopy times, as well as the use of fewer guidewires, balloons, and stents, which affect the affordability and long-term prognosis of the CTO procedure. Polymeric and moderate stiff tip guidewires showed a superior performance in crossing the CTO segment. The choice of the Pilot brand in lesions with J-CTO scores of 0–1 would increase the probability of crossability with the initial guidewire. In such lesion types, non-polymeric stiff guidewires should not be used as the initial guidewire.

In each CTO procedure, the use of 1–3 guidewires can be accepted as an economical and rational use of guidewires. In each procedure, a standard soft tipped or polymer-jacketed guidewire was used to place the microcatheter. Then, special CTO guidewires replaced the initial standard wire for lesion penetration. In the final stage, the standard wire was exchanged with the CTO wire once again to perform the balloon and stenting procedure. Special CTO wire should be used just for lesion penetration. This strategy necessitates at least 2–3 wires for each procedure. In our cases, the average number of guidewires was < 4 in lesions with J-CTO scores of 0–1. We can conclude that the use of > 4 guidewires with the antegrade technique in lesions with J-CTO scores of 0–1 was not rational, and it can be defined as overuse. The crossability of the polymeric soft and moderate stiff tip guidewires was not so good in lesions with J-CTO scores > 2. For this more complicated lesion, stiff tip non-polymeric guidewires should be used. The Gaia brand showed a more significant crossability performance in lesions with J-CTO scores > 2. The distribution of the Fielder brand was similar between all J-CTO scores. But there was a small detail in the performance of Fielder guidewires. In the lesions with J-CTO scores of 0–1, the crossability of the Fielder guidewire as an initial choice was higher, and the single-wire technique was usually preferred. In the more complex cases, other techniques, particularly the step up and step down strategy, were preferred. Lesions were modified with multiple guidewires, including non-polymer stiff guidewires and Fielder guidewires, that were used to just jump to the true lumen. Thus, a similar distribution of the Fielder brand as a final guidewire did not indicate the strong crossability of lesions with J-CTO scores > 2.

The EuroCTO or CASTLE score was defined with the help of the prospective EuroCTO registry [11]. This novel scoring chart was compared with the J-CTO score, and it was found to be superior in more complex cases. We performed a J-CTO score-like analysis also for the EuroCTO score. Our result was comparable for both scoring methods. Lesions with EuroCTO scores of 0–1 showed a result similar to lesions with J-CTO scores of 0–1. The average guidewire number was < 4, and more than 50% of the lesions were crossed with the initial guidewire. Polymeric moderate stiff tip guidewires showed a superior performance in crossing the CTO segment as an initial guidewire choice. However, final wire brand, composition, and stiffness did not differ among the EuroCTO score group. Nonetheless, we can state that stiffer tip guidewires were preferred with higher EuroCTO scores. As a limitation, there were just four lesions with EuroCTO scores of 5–6, which significantly reduced the statistical analysis for very complex lesions. Non-polymeric moderate stiff tip guidewires were used more commonly in lesions with EuroCTO scores 1–4 as a final guidewire compared to the initial wire type. Our data confirmed that the polymeric moderate stiff tip Pilot brand was a good choice for lesions with EuroCTO scores 0–1.

Our results strongly confirmed that the use of CTO scoring would influence our technique in a favorable way, which directly affect the initial guidewire choice. Single-wire escalation is associated with the use of fewer guidewires. For the easy and intermediate difficulty CTO lesions, single-wire crossing should be the initial choice for rational guidewire usage. Other techniques including parallel wire, step up-step down, and dissection and re-entry were associated with a very low probability of penetration of the lesion with the initial guidewires. However, the parallel wire technique offered more rational guidewire use. The average number of guidewires used was lower when comparing to step up-step down and dissection and re-entry techniques. Polymeric guidewires were more successful in single-wire escalation. Non-polymeric guidewires should be chosen in the other techniques. According to lesion modification during wire escalation and drilling, optimal guidewire exchange should be performed. For the dissection and re-entry technique, polymeric guidewires should be preferred to jump into the true lumen.

The use of new generation guidewires would increase the success rate. However, the affordability of new generation guidewires would limit their optimal preference. The Fielder brand is one of the most commonly preferred soft polymeric guidewires. We usually preferred the Fielder XT series rather than the new generation Fielder XT-A. This choice may overshadow the crossability of the Fielder brand. The new generation Fielder XT-A series has an additional composite core technology in the guidewire tip, which enhances crossability. We used Fielder XT-A series in only four cases, which did not show statistically significant superiority. On the other hand, the new generation non-polymeric guidewires are preferred compared to the older series. The Miracle brand is a well-known non-polymeric guidewire from Asahi Intecc. The new generation Gaia brand with composite core technology has replaced the Miracle series. In our experience, the Gaia brand was preferred over the Miracle series, which would directly affect the success rate. On the other hand, we used first-generation Gaia 1-2-3 brands. The newly released Gaia Next series, which has Xtrand coil technology, was not used in any of the cases. For more complex lesions that necessitate a stiff tip load, Confianza 9 was preferred over the Confianza Pro series, which possesses a thinner tip and has a more slippery hydrophilic coating. Moreover, we also used Gladius guidewires as a last resort in certain cases. The Gladius brand is a new generation peripheral guidewire, which has a durable balanced tip composition. It has relatively superior crossability for intermediate and difficult CTO lesions. Although it is designed for peripheral interventions, it can be used for coronary CTO procedures in selected cases (as a rescue solution). On the other hand, the newly released Gladius MG guidewire series was not used due to its unavailability.

Preference for new generation guidewires would lead to more successful procedural results [21, 22]. However, the experience of the operator was also just as important as technology [23]. Our case series, newly released guidewires were used in a few cases. Nonetheless, our procedural success rate was above the average (approximately 90%). Each operator has a special relationship with guidewires, and each has their favourite guidewire for different occasions. Experienced operators can predict the behavior of the guidewire in each of the different lesion compositions. Indeed, all operators want to perform CTO procedures with newly released technology. Unfortunately, it is not possible in most cardiac centres located in Turkey. For this reason, the operator should know the composition and behavior of all the guidewire brands to choose guidewires in a rational manner.

### Limitations

The first limitation is a relatively small sample size. For assessing newly released biotechnology, we analyzed the recent past period of time. So, our case sample is relatively small. All the procedures were performed by the same CTO team, which raises a question about the personal preference of the guidewires. Although the procedures were performed by the same team, the cases were collected from different hospitals, which have their own catheter laboratory and independent purchasing department. So, the CTO operators preferred the guidewire according to its availability in the catheter laboratory.

## Conclusion

Adequate strategy and logical guidewire choice are the key for successful CTO interventions. Although our success rate for the procedures was higher, the use of newly released guidewire technology was relatively limited. This result showed that it would take some time to get use to the newly released biomaterials in clinical practice. Classification of the CTO lesion by J-CTO and EuroCTO scores would influence the choice for strategy and guidewire preference. In lesions with J-CTO scores of 0–1 and EuroCTO scores of 0–1, the single-wire crossing technique was associated with the use of fewer guidewires. Most of these lesions could be crossed by the initial guidewires. Polymeric, moderate stiff tip guidewires, particularly the Pilot brand, had superior crossability as an initial guidewire. The same initial-final guidewire strategy also had fewer balloons used, shorter stent length, shorter fluoroscopy duration, and decreased amounts of contrast used. However, the same initial-final guidewire strategy was not successful in difficult and very difficult lesions (J-CTO score > 2, EuroCTO score > 2). In such groups, new innovative non-polymeric guidewires, particularly the Gaia brand, seemed to be superior with regard to lesion penetration.

## Data Availability

The datasets used and/or analyzed during the current study are available from the corresponding author on reasonable request.

## Abbreviations

CTO: Chronic total occlusion
J-CTO: Japanese chronic total occlusion registry
PCI: Percutaneous coronary intervention
TIMI: Thrombolysis in myocardial infarction
SPSS: Statistical Package for the Social Sciences
CI: Confidence interval
OR: Odds ratio
HT: Hypertension
DM: Diabetes mellitus
MI: Myocardial infarction
CABG: Coronary artery bypass graft
